# Bridging the gap between diabetes care and mental health: perspectives of the Mental health IN DiabeteS Optimal Health Program (MINDS OHP)

**DOI:** 10.1186/s12902-021-00760-3

**Published:** 2021-05-08

**Authors:** Lucienne Ferrier, Chantal F. Ski, Casey O’Brien, Zoe Jenkins, David R. Thompson, Gaye Moore, Glenn Ward, David J. Castle

**Affiliations:** 1 Department of Psychiatry, University of Melbourne, Melbourne, Australia; 2 Integrated Care Academy, University of Suffolk, Ipswich, UK; 3 School of Nursing and Midwifery, Queen’s University Belfast, Belfast, UK; 4 Mental Health Service, St. Vincent’s Hospital, Melbourne, Australia; 5 Department of Endocrinology, St. Vincent’s Hospital, Melbourne, Australia

**Keywords:** Type 1 diabetes mellitus, Mental health, Optimal Health Program

## Abstract

**Background:**

Mental health problems are highly prevalent in people with type 1 diabetes mellitus (T1DM), which adversely impact physical health and quality of life. This study aimed to explore the experiences of people with T1DM who had completed the Mental health IN DiabeteS Optimal Health Program (MINDS OHP), a novel intervention developed to bridge the gap between physical and mental health care.

**Method:**

Participants with T1DM were invited to take part in a focus group or semi-structured interviews. Participants were recruited from outpatient and community settings. The focus group and interviews were audio-recorded and transcribed verbatim. Thematic content analysis was used and identified themes were cross-validated by researchers and member-checked by participants.

**Results:**

Ten people with T1DM were included. Two key themes emerged: ‘MINDS OHP experiences’ and ‘lived experiences of diabetes’. MINDS OHP experiences included five sub-themes: program benefits, follow-up and timing, suggested improvements, collaborative partners, and materials suitability. Lived experiences also included five sub-themes: bridging the gap between mental and physical health, support networks, stigma and shame, management intrusiveness, and adolescence and critical life points.

**Conclusions:**

The MINDS OHP for people with T1DM was generally well received, though study findings suggest a number of improvements could be made to the program, such as including family members, and consideration being given to its routine early inclusion in diabetes management, ideally in primary care.

**Supplementary Information:**

The online version contains supplementary material available at 10.1186/s12902-021-00760-3.

## Key points

Care for people with T1DM often fails to adequately address the needs of family members and adolescents, and early intervention and inclusion of families in the diabetes care process has the potential to improve diabetes outcomes and experience.

People with T1DM appear to appreciate novel interventions such as the MINDS Optimal Health Program which bridge the diabetes and mental health care divide, and welcome consideration being given to the inclusion of diabetes educators in their routine care.

## Background

Individuals with type 1 diabetes mellitus (T1DM) are at increased risk of poor mental health, particularly depression and anxiety [1–3]. The intrinsic link between mental health and T1DM is frequently seen in primary care settings, but often not addressed specifically in routine clinical practice [4]. The experience of T1DM may negatively affect mental health, while mental health reciprocally influences metabolic control, and suboptimal glycaemic control may result in adverse long-term health consequences and reduced quality of life [3, 5–7].

Additionally, T1DM has a notable impact upon family members of individuals with the condition, particularly parents and caregivers [8–10]. Family members are likely to experience increased stress due to the responsibility of co-managing the burdensome treatment regimen [9–11]. Diabetes-related stress experienced by families often becomes accentuated during the adolescent period, with particular concerns about long-term health consequences [2, 5, 12].

In an attempt to address diabetes-specific aspects of care we developed the Mental health IN DiabeteS Optimal Health Program (MINDS OHP), a psychoeducational self-empowerment intervention [13]. It has a modular format encompassing an 8-week intervention, one module per week, and a booster session (Table 1). The original OHP demonstrated effectiveness for people attending mental health services [14]. The MINDS OHP incorporates supplementary diabetes-relevant material [13, 15] and targets the psychological impact of T1DM, with the aim of empowering individuals to make informed health-related decisions and improve overall health and wellbeing. To ascertain whether the program is fit for purpose and addresses the specific needs of people with diabetes, participant feedback is essential. Qualitative research methods are the most appropriate way of exploring issues such as patients’ motivations, perceptions and expectations [16].

**Table 1:** The Mental Health IN DiabeteS Optimal Health Program (MINDS OHP)

Session title	Objectives	Content
1. Introduction to OHP model	1. Define optimal health 2. Consider how our behaviour influences health 3. Self-assessment 4. Introduce Health Plans 1–3	Considers six domains; mental, emotional, social, occupational, physical and spiritual health. Provide opportunity to explore and understand current self-management behaviour and satisfaction with day-to-day functioning. Personal and family beliefs about diabetes
2 & 3. “I-Can-Do” Model	1. Complete own “I-Can-Do” Model, 2. Identify own strengths, vulnerabilities 3. Understand Health Plan 1	Sessions 2 and 3 introduce ‘I Can Do’ model, which encompasses health plans exploring the participant’s strengths and vulnerabilities, and anticipates effects of crises and developing strategies to overcome these Balancing hope with reality – coping with diabetes complications. Exploring how anxiety affects diabetes and vice versa.
	1. Identify stressors, including those linked to diabetes 2. Explore early warning signs 3. Stress management strategies: Health Plan 2	
4. Medication and lifestyle	1. Identify +/− aspects of medication, medication monitoring 2. Understand value of metabolic monitoring and healthy lifestyle	Lifestyle and physical health management, impact of healthy diet and exercise. Effective use/self-management of medication, any side-effects. Adjusting to diabetes treatments including medication, insulin delivery methods, diet and lifestyle changes.
5. Collaborative partners (CP) and strategies	1. Understand importance of CPs 2. Identify/plan roles of people/supports as CPs 3. Make Health Plan 3 and Eco Map	Develop an ‘Eco Map’ detailing key partnerships and supports in the participant’s network and community (e.g. GP, other healthcare supports, family). Identify gaps in support/care and make plans to overcome any barriers to engaging peer and community support for living with diabetes.
6. Change enhancement	1. Understand the Wellbeing Timeline 2. Explore ‘Sub-optimal Health’ and episodes of illness 3. Revisit Health Wheel, meaning of change	Change enhancement by tracking health fluctuations across time and establishing new proactive avenues for change. Revisit Health Wheel: Visioning and Goal setting. Exploring how problem-solving can support diabetes self-management.
7. Visioning and goal setting	1. Identify change and its meaning 2. Explore key steps in problem solving and principles of goal setting	Discusses goal setting via creative problem solving and planning in diabetes, guided by own priorities. Also allows reflection of what is useful in any future crises.
8. Maintaining wellbeing	1. Understand Health Plans 1–3, Health Journal 2. Introduce/plan booster session	Reviews well-being maintenance and sustainability by acknowledging any progress made towards goal, exploring concept of using rewards to improve progress.
Booster How is my health now?	1. Revision, catch up, consolidation of progress 2. Review Health Plans 1–3 3. Celebrate achievements	Review Health Plans 1–3, understand how Health Plans maintain OH. Celebrate achievements, reflect on experience.

We therefore, as part of a larger study evaluating effectiveness of the program [13, 15], conducted a qualitative study to explore the experiences of people with T1DM who had completed the MINDS OHP.

## Methods

### Study design

 We conducted a qualitative study with a convenience sample of people with T1DM who were invited to participate in a focus group or, if unable to attend, an individualized face-to-face semi-structured interview. Participants were considered eligible for inclusion if they had a confirmed diagnosis of T1DM, were 18 years of age or over, and were able to converse in English without an interpreter [13]. Participants were excluded if they had a developmental disability or amnestic syndrome impairing their ability to learn from the intervention, or a comorbid serious acute medical illness defined by the treating physician.

For the purpose of this study, an interview schedule was prepared to guide questions and prompts about participants’ experiences (e.g. benefits, negative aspects, extent of support, suitability) of the MINDS OHP (Additional file 1).

### Recruitment and data collection

Full details of the MINDS OHP trial protocol have been published elsewhere [13]. In brief, all participants had completed on a 1:1 basis the MINDS OHP with a trained facilitator. The trial setting was a tertiary hospital in a metropolitan area of Melbourne, Australia, although participants were recruited from outpatient clinics and community settings through advertisements. Those who completed the MINDS OHP were invited to participate. After a positive response, interviewers introduced themselves over the phone and an appointment was arranged.

LF, a final year medical student, with experience in interviewing, conducted the focus group and interviews at a clinic during March to May, 2019. A sample size of 10–14 was considered sufficient to reach data saturation and understand participants’ experiences in thematic content analysis [17]. For studies such as this 6–10 participants are recommended for interviews and 2–4 for focus groups [17].

Participants were invited to take part in a focus group or, if unable, a scheduled semi-structured interview.

### Data analysis

The focus group and individual interviews were tape-recorded, transcribed and data subjected to thematic analysis. Identified themes were cross-validated by researchers and member-checked by participants. Interviews were recorded using a digital voice recorder. Interview recordings were saved on a computer drive to which only two researchers had access, with the recording device stored in a locked cabinet. Additionally, ZJ took notes in the interviews as a backup for recordings and to observe nonverbal nuances [18]. Neither LF or ZJ were involved in the clinical delivery of MINDS OHP.

**Fig. 1:**
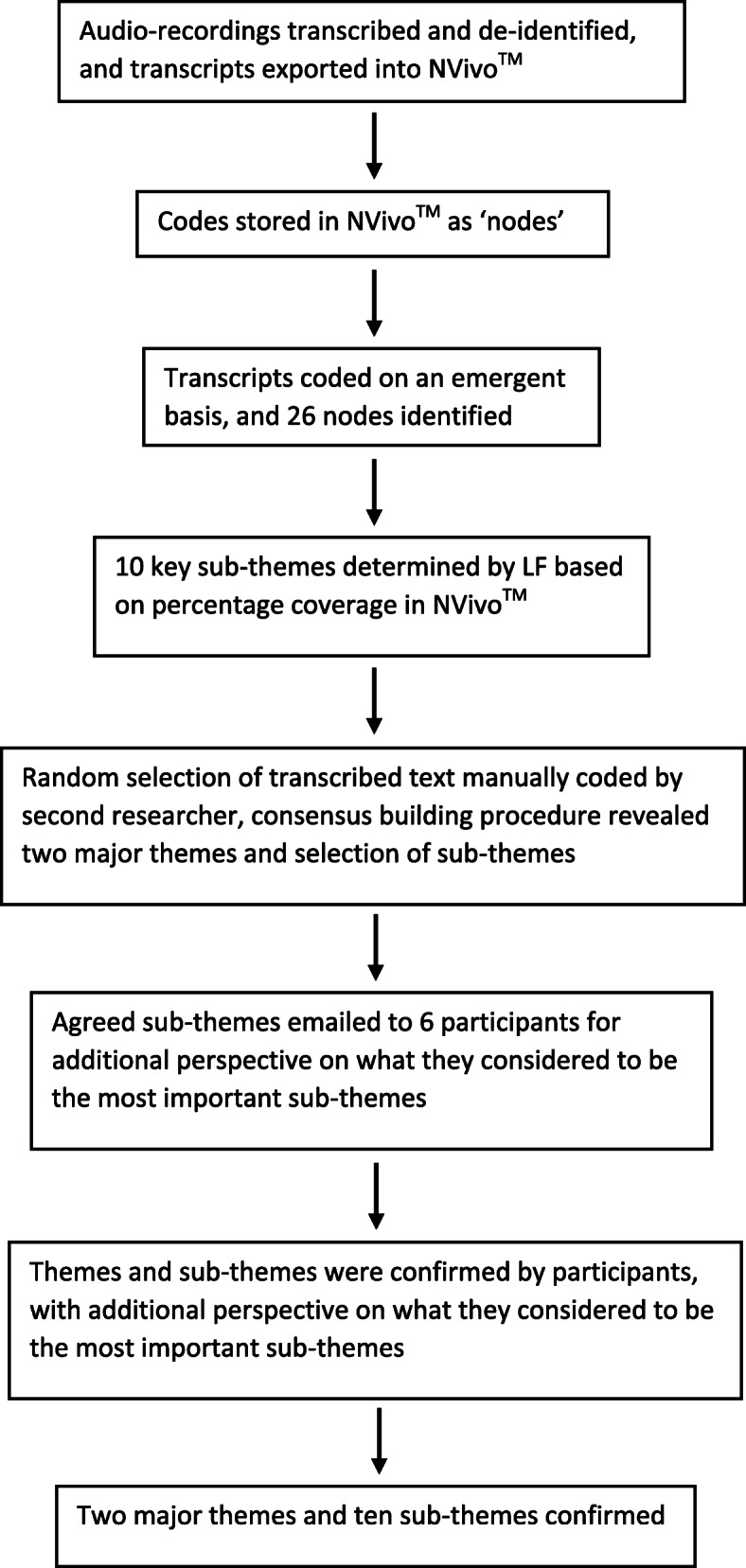
Thematic data analysis

Themes were determined on an emergent basis to prevent data being influenced by pre-ordained ideas, and to attain lived experiences from participants with minimal facilitator prompting. Thematic analysis was undertaken using NVivo™ software [18]. Inductive thematic analysis was used to code the data without fitting it to preexisting ideas, reducing researcher bias [19]. The co-facilitator’s notes were reviewed and utilised to supplement the information gained from interview recordings, particularly for observations on non-verbal communication. Consensus building and member checking were also employed for objective categorization (Fig. 1).

Within themes, we looked for differences and similarities between participants. LF and ZJ discussed final codes and themes. Quotes were extracted to illustrate aspects of themes using participants’ own words.

### Ethics

 The study was approved by the Human Research Committee of St. Vincent’s Hospital Melbourne (HREC-A 036 − 14) and conducted in accordance with the Declaration of Helsinki.

## Results

Informed consent was obtained from 14 individuals, though one subsequently declined the interview, one withdrew due to personal circumstances, and two did not respond to repeated contact attempts, leaving 10 participants.

 Five of the 10 participants took part in a focus group and the remaining five were interviewed individually, face-to-face. LF conducted all the interviews.

The 10 participants (6 females; 4 males) were aged from 25 to 70 (mean 47) years.

Two themes (MINDS OHP experiences and lived experience with diabetes), each with five sub-themes (program benefits, timing and follow-up, suggested improvements, collaborative partners, materials suitability; mental health, support networks, stigma and shame, management intrusiveness and complexity, adolescence and critical life points) respectively emerged from the data (Table 2).

**Table 2:** Patient perspectives and experiences of the Mental Health IN DiabeteS Optimal Health Program (MINDS OHP): themes and subthemes

Themes:	MINDS OHP experiences	Lived experiences with T1DM
Sub-themes:	Program benefits	Mental health
	Timing and follow-up	Support networks
	Suggested improvements	Stigma and shame
	Collaborative partners	Management intrusiveness
	Materials suitability	Adolescence and critical life points

### MINDS OHP experiences

Participants [P] discussed their overall experiences from the program, with five sub-themes arising.

### Program benefits

Feedback about the program was highly positive throughout the interviews. Most participants emphasised the relevance, educational value, and broad perspective of the program. For example, one [P1] stated that the program “turned my life on its head”, “made me refocus” and think about their diabetes “more holistically”.

### Timing and follow‐up

Most participants appreciated the program’s timing and novelty, with one [P2] suggesting that ideal timing of program delivery should be “not long after you’re diagnosed”, and another [P3] that it should be from “day 1” of diagnosis.

### Suggested improvements

Participants recommended ways in which the program could better support people with T1DM, including diabetic educators being involved in session formulation and facilitation due to their unique perspective and expertise, and better training facilitators in diabetes management. One participant [P4] recommended that “a list of psychiatrists” or other mental health professionals who were familiar with the program would be a valuable referral source; another [P5] that more health professionals should be “aware that the program is on offer”.

### Collaborative partners

Participants praised the support and flexibility of program facilitators in adapting to their individual circumstances. Some participants emphasised that the program should be offered to and by health professionals involved in T1DM care, as one [P6] reflected that “no health professional had ever brought this up with me” prior to this program. A participant [P5] suggested that the program should encourage attendance of a carer or family member for at least one session, and that “a program for carers” would be appreciated; another [P3] recommended that “type 1’s and their partners…should be counselled at the same time”.

### Materials suitability

Mode of delivery and access to the program were consistently mentioned as areas for improvement. Some participants recommended that program outreach could be improved via phone or videoconferencing technology: one participant [P7] suggested that the program could “meet them in their homes” to improve accessibility and engagement, and another [P6] reflected upon the geographical “barrier” to participation in regional areas. Most participants commented positively on the program workbooks and “homework” exercises, although some described the exercises as being “generic” rather than diabetes-specific.

### Lived experience with T1DM

Participants emphasised their lived experience with T1DM, which arose as a distinct theme despite some overlap with their MINDS OHP experiences. Five sub-themes were identified.

### Mental health

Participants emphasised the importance of the program in supporting wellbeing. Many described the mental health impacts they experienced due to the complexity and intrusiveness of diabetes management, alongside the reciprocal impacts of stress upon their diabetes. One participant [P2] described the “stress and trauma” often experienced by people with diabetes, and another [P8] identified that “outside stresses, they make a mess of your diabetes”. Furthermore, participants consistently raised the concept of *bridging the gap* between mental health and diabetes care, and the importance of diabetes educators as astute patient advocates, with one participant [P2] describing diabetes educators as being “a therapist and medical help at the same time”, and another [P4] as “gold” due to their unique understanding of management complexities.

### Support networks

Participants consistently emphasised the importance of peer connection and support in living with T1DM, particularly the unique understanding provided by peers with diabetes. A participant [P4] reported “it’s a very big thing, that peer support”, and another [P8] reflected “nobody knows what it’s like except for those who’ve got it”. A participant [P2] described family support as “integral” to T1DM management, particularly spouses and parents. All participants reflected on their earlier experiences of diabetes support, including parents often being responsible for managing their child’s T1DM and being their “greatest advocate and supporter” [P3], and having needed additional support themselves. They felt that the program could provide greater education and support for family members.

### Stigma and shame

 Participants identified stigma as a key stressor in T1DM, with one [P6] describing T1DM as a disease that people were ashamed to disclose due to “numerous negative connotations”. Participants also emphasised the perceived ignorance of T1DM by society and family members, and feeling ashamed or obligated to internalise their diabetes concerns. A participant [P5] reflected “the ignorance out there, it’s unbelievable” and criticised the media for circulating misinformation about T1DM, and other participants agreed that wider awareness and education is needed to address this. Participants explored the importance of T1DM advocacy and individualised care, and one [P8] reflected “nobody…is a better advocate or a better source of information that someone who’s got it”. Many participants expressed feeling unheard and dismissed by health professionals, and the need for clinicians to validate patients’ concerns and help them understand their T1DM.

### Management intrusiveness

Participants identified that the constant vigilance associated with diabetes management contributes to diabetes-related stress. They reported that constantly monitoring blood sugars exacerbated stress and anxiety in themselves and their families, with one participant [P1] identifying that these mental health impacts “[weren’t] *just* diabetes, but it was everything else that came with it”. Another participant [P6] reflected upon a number of “really nuanced scenarios”, such as exercise and stress, that may adversely impact diabetes management.

### Adolescence and critical life points

Participants identified adolescents as being particularly vulnerable to diabetes-related distress and adverse health consequences, and participants reflected on their own adolescent experiences. A participant [P1] described the importance of addressing “critical time points” such as initial diagnosis or during adolescence, particularly given the difficulties for adolescents adjusting to their diagnosis, and being able to “process their feelings and emotions” alongside their physical health. Participants identified that the program could be an appropriate intervention at this particular life transition point.

## Discussion

This qualitative study of the MINDS OHP identified two key themes - program experiences and lived experiences with T1DM - which have implications for informing program development, reach and impact. Although the sample may appear relatively small, it was judged to be of sufficient size for this type of study [17]. However, we would recommend that the study be replicated in other settings with a larger sample.

 We found that though the program was generally well regarded by participants there were some areas requiring improvement, especially concerning the needs of family members and adolescence as a critical time-point. These findings highlight a gap in routine T1DM care, and provide valuable insights into how the MINDS OHP could be improved to best fill the gap. They also have broader implications for the integration of interventions such as MINDS OHP into routine and earlier clinical practice, ideally in primary care, and the role of health professionals such general practitioners and diabetes educators.

Participants praised the broader perspective offered by the MINDS OHP, consistent with the literature regarding paucity of comprehensive interventions addressing mental health in T1DM [2]. However, there were also some logistical issues raised about the program, such as access from rural and remote areas, and suggestions that sessions could be facilitated via phone or home visitation to improve outreach. This has since been addressed, with phone-based sessions being offered to participants, reflecting the program’s ability to continually adapt according to feedback. Similarly, participants suggested that a list of mental health professionals who were familiar with diabetes could be made available, which has also now been addressed. Some participants suggested enhancement of the program by involving diabetes educators in session formulation, and better training facilitators in T1DM management.

 An unexpected facet of the interviews was the participant use of the focus group as a peer support environment, and participants suggesting that the program itself could include a group component to enable this. This emphasis on psychosocial support is consistent with the literature on T1DM [1], and perhaps reflects a tendency for participants to discuss their experiences following program completion, or to communicate their individual stories to contextualise their program experiences. In regard to lived experiences, the interlinking of mental health and diabetes was repeatedly emphasised, consistent with the literature [3]. Participants highlighted the importance of bridging the gap between mental health care and diabetes care through targeted psychoeducation sessions and diabetes educators, again an issue consistent other findings [20]. Participant feedback highlighted the rich diversity of lived experiences among those with diabetes, and reinforced the importance of viewing those with T1DM as unique individuals, each within their own social context.

Stigma was also identified as a key issue, with associated shame and fear of disclosure, due to misperceptions of T1DM and moralistic societal ideas of personal guilt impressed upon them. This finding echoes those from other studies that stigma is a notably negative experience of many people with diabetes [1], though many participants also acknowledged that societal awareness of T1DM has improved over time.

Our findings are in agreement with other studies [8] that found adolescents experience unique challenges with diabetes in the transition to adulthood as they navigate educational and relational milestones. Participants agreed that teenagers were likely to be overwhelmed and stressed by their diabetes diagnosis and management [7], and expressed that they would have liked the MINDS OHP to have been available soon after they were diagnosed, but also that the program could be offered flexibly according to individual needs rather than at a standardised time after diagnosis.

A dual aim of this study was to explore whether the program be adapted to support family members. Most participants praised the support they received from their families, and identified parents and partners as key supports for people with diabetes, with parents being particularly impacted by management issues and associated stress related to their child’s T1DM, consistent with other findings [7]. This reflected the perceived need for greater support for family members already identified within the literature, and which participants believed could be provided by the program [9, 10].

Participant experiences regarding family members also highlight a continuing clinical gap. The literature notes that family members are often impacted by their loved one’s T1DM, but their experiences are often not addressed routinely. This study therefore provides specific suggestions for how the program could address the needs of family members, including supportive counselling, education, and involvement in selected program sessions.

Finally, the suggestions regarding extended follow-up could assist in reinforcing learning from the program, whilst the potential for group discussion could serve as an important psychosocial support system for those with T1DM. Suggestions to involve family members and health professionals in sessions could also assist in wider education for the support networks of people with diabetes. As a result, those participating in the MINDS OHP could be more empowered and supported to take responsibility of their health and control of their T1DM, with the likely long-term benefits to overall health and quality of life.

### Strengths and limitations

Strengths of this study include the use of qualitative research methods which are becoming more recognized and valued in diabetes behavioural research [1, 16, 19]. The use of such research methods has elicited a breadth of participant experiences, with resultant data richness and diversity. The three-pronged validation process used in the data analysis procedure also ensured appropriate rigour and minimised potential bias. However, variance in data collection methods - focus groups or interviews - may have influenced the type of data obtained. Integrating participant responses from both methods may have led to a combination of in-depth and real-world data not acquired by using one method alone. For the purpose of this study, the choice of the two methods was determined by the capacity of participants to physically attend either. It is also unclear whether these findings would be applicable to other ethnic and cultural groups, as the participants in this study were ethnically homogeneous. An alternative form of qualitative analysis such as framework analysis could have been used, but we decided against this due to the potential for bias of reported experiences.

The small sample size may be perceived as a limitation, though we have justified it as sufficient for this type of study.

This study provides pointers for future research, including potential adaptation of the program to incorporate families and carers, and exploring the specific impact of T1DM upon adolescents and how MINDS OHP could address this population more overtly. Other suggestions for future research include increasing involvement of diabetes educators in program conception and facilitation. By doing so, MINDS OHP could be offered as a routine component of a holistic package delivered early after the diagnosis of T1DM, ideally in primary diabetes care. This could include the program being introduced to newly-diagnosed T1DM patients by their general practitioner, probably with input from a diabetes educator and practice nurse to review and monitor progress.

## Conclusions

The MINDS OHP was generally well received as a psychoeducational intervention for individuals with T1DM. However, findings from this study highlight gaps, including the failure to address more overtly the needs of family members and adolescents, particularly early on. It is recommended that bridging this gap will contribute to improved development, reach and impact of the program. Consideration should be given to the inclusion of the program in the clinical management of diabetes, preferably in primary care.

## Supplementary information

Additional file 1. Interview Guide. The Mental Health IN DiabeteS Optimal Health Program (MINDS OHP) Interview Guide.

## Data Availability

The data transcripts in anonymized form are available from the corresponding author.

## Abbreviations

T1DM: Type 1 diabetes mellitus
MINDS OHP: Mental Health IN DiabeteS Optimal Health Program
