# Supplementary material for: Bridging the gap between diabetes care and mental health: perspectives of the Mental health IN DiabeteS Optimal Health Program (MINDS OHP)
Source: BMC Endocr Disord. 2021 May 8;21:96. doi: 10.1186/s12902-021-00760-3 (PMC8105945; doi:10.1186/s12902-021-00760-3)
Supplement: Supplementary file 1 — Interview Guide. The Mental Health IN DiabeteS Optimal Health Program (MINDS OHP) Interview Guide. [file 12902_2021_760_MOESM1_ESM.docx]

**Additional File**

The Mental Health IN DiabeteS Optimal Health Program (MINDS OHP) Interview Guide

1. Describe your experience of being involved in the program?
2. Have there been any benefits of being involved in the program?
3. Have there been any negative aspects of being involved in the program?
4. Please tell us about the level of support you received from the project staff. Was this sufficient?

Prompt: What would you have liked to be done differently in relation to the program?

a) Do you think that it could also address the issues of family members? If so, how? b) Would a program like this have been helpful when you were first diagnosed?

1. Please comment on the suitability of the materials for people living with diabetes.
2. We’ve received feedback that some participants would like to stay involved with the program in some way. What are your thoughts about this, and about the possibilities we could offer for further follow-up?
3. Is there anything else you would like to tell us about your involvement in the program?
